# Supplementary material for: Lived experiences of recovered COVID-19 persons in Nigeria: A phenomenological study
Source: PLoS One. 2022 Aug 15;17(8):e0268109. doi: 10.1371/journal.pone.0268109 (PMC9377583; doi:10.1371/journal.pone.0268109)
Supplement: S2 File — (DOCX) [file pone.0268109.s002.docx]

Project Title: COVID-19: A qualitative investigation of the experience of recovered patients in Nigeria

**In-depth Interview Guide**

**Ice-breaker**

I am wondering what will happen to our economy if the price of crude oil remains low. What do you think?

**The possible mode through which the virus was contacted**

1. How do you think you contracted the virus?
2. Did you follow COVID-19 preventive practices such as mask, handwashing, social distancing?

Probe for travel history, and other modes

**Experience with testing, discovery, isolation, and treatment**

1. Did you have any of the symptoms of coronavirus? How severe?

Probe for the specific symptoms

1. How did you get tested?

Probe for where the test was done

1. How was the result delivered to you?

Probe for who delivered the result - health provider

1. What was your reaction to the result?
2. How was your experience in the isolation centre?
3. How did you cope with the entire experience -virus, boredom, symptoms?
4. Did you at any time think about death, and what were your fears?

Probe for fear and anxiety, and concern for family members

Probe for provider-patient relationship, patient-patient relationship, feeding, environment, visits from relatives, duration of stay, use of phones etc.

1. What were you treated with?
2. What traditional medicine did you use and how effective?

Probe for specific types of drugs, supplements, and any other thing

**Social and financial effects**

1. What about the financial cost?

Probe for who paid the bills, other costs

1. Would you say the experience has affected the way people relate to you?

Probe for more details on the response; experience of stigma, discrimination

1. How about the way you relate to people?

Probe for what has changed

**Perception about management and treatment of coronavirus and recommendation**

1. Would you say the management and treatment of coronavirus in your case was effective?

Probe for reasons for the response; probe for perception about management and treatment in general.

1. In all, what advice would you give for better prevention and control of coronavirus

Probe for specific advice to policymakers, health providers, the public

1. Any other issue you would like to talk about that we have not discussed.

Thank you.
