# Supplementary material for: Lived experiences of recovered COVID-19 persons in Nigeria: A phenomenological study
Source: PLoS One. 2022 Aug 15;17(8):e0268109. doi: 10.1371/journal.pone.0268109 (PMC9377583; doi:10.1371/journal.pone.0268109)
Supplement: Attachment — Submitted filename: Rebuttal Letter.docx [file pone.0268109.s003.docx]

Dear Editor,

Re: PONE-D-21-19514
Lived experiences of recovered COVID-19 persons in Nigeria: A phenomenological study

Thank you for considering our article for publication in your journal. We have now responded to the editorial, and reviewer’s comments. Please, see the point-by-point response below.

Your Sincerely,

Friday E. Okonofua

Corresponding Author

Point-by-point response

Journal requirements:

1. Please ensure that your manuscript meets PLOS ONE's style requirements, including those for file naming. The PLOS ONE style templates can be found at

Response: Our manuscript meets the style requirements.

2. In the Methods section, please provide additional information regarding the interview guide development process, including the theories or frameworks which were employed. And please include a copy of the interview guide as supporting file.

Response: This has been addressed in the methods, and the guide is uploaded as a supporting file.

3. In your Data Availability statement, you have not specified where the minimal data set underlying the results described in your manuscript can be found. PLOS defines a study's minimal data set as the underlying data used to reach the conclusions drawn in the manuscript and any additional data required to replicate the reported study findings in their entirety. All PLOS journals require that the minimal data set be made fully available. For more information about our data policy, please see <http://journals.plos.org/plosone/s/data-availability>.

Upon re-submitting your revised manuscript, please upload your study’s minimal underlying data set as either Supporting Information files or to a stable, public repository and include the relevant URLs, DOIs, or accession numbers within your revised cover letter. For a list of acceptable repositories, please see <http://journals.plos.org/plosone/s/data-availability#loc-recommended-repositories>. Any potentially identifying patient information must be fully anonymized.

Important: If there are ethical or legal restrictions to sharing your data publicly, please explain these restrictions in detail. Please see our guidelines for more information on what we consider unacceptable restrictions to publicly sharing data: <http://journals.plos.org/plosone/s/data-availability#loc-unacceptable-data-access-restrictions>. Note that it is not acceptable for the authors to be the sole named individuals responsible for ensuring data access.

We will update your Data Availability statement to reflect the information you provide in your cover letter.

Response: Access to the data associated with this article is restricted for ethical reasons due to the potentially identifying and sensitive information about the participants who were COVID-19 patients. However, on request from the corresponding author, some of the data may be shared.

4. Please review your reference list to ensure that it is complete and correct. If you have cited papers that have been retracted, please include the rationale for doing so in the manuscript text, or remove these references and replace them with relevant current references. Any changes to the reference list should be mentioned in the rebuttal letter that accompanies your revised manuscript. If you need to cite a retracted article, indicate the article’s retracted status in the References list and also include a citation and full reference for the retraction notice.

Response: The references have been updated. There are no omitted references

**Reviewers' comments:**

Reviewer #1: Some figures need to be inserted for example the Ct or MRI of the patients, or x-ray of their chest. Also, some laboratory tests should be presented in a table to be very attractive article with statistical analysis.

Response: Thank you. However, we are unable to insert the suggested figures because of the following: 1) The study is a qualitative study, and statistical analysis is not appropriate, 2) The Ct or MRI of the patients or their x-rays are confidential documents belonging to the different hospitals, and isolation centre where the respondents were treated. The are not accessible to us.
