# Supplementary material for: Lived experiences of recovered COVID-19 persons in Nigeria: A phenomenological study
Source: PLoS One. 2022 Aug 15;17(8):e0268109. doi: 10.1371/journal.pone.0268109 (PMC9377583; doi:10.1371/journal.pone.0268109)
Supplement: Attachment — Submitted filename: Response to Reviewers.docx [file pone.0268109.s004.docx]

April 14, 2022

Dear Editor,

Re: PONE-D-21-19514R1
Lived experiences of recovered COVID-19 persons in Nigeria: A phenomenological study

Thank you for considering our article for publication in your journal. We have now responded to the editorial, and reviewer’s comments. Please, see the point-by-point response below.

Your Sincerely,

Friday E. Okonofua

Corresponding Author

Point-by-point response

Journal Requirements:

Please review your reference list to ensure that it is complete and correct. If you have cited papers that have been retracted, please include the rationale for doing so in the manuscript text, or remove these references and replace them with relevant current references. Any changes to the reference list should be mentioned in the rebuttal letter that accompanies your revised manuscript. If you need to cite a retracted article, indicate the article’s retracted status in the References list and also include a citation and full reference for the retraction notice.

Response: The reference list is correct and complete. We did not cite any article that has been retracted.

Reviewers’ comment

Reviewer #2

How did the participants grant a written informed consent when participation is virtual? Also, the authors should include the ethical review number granted by the Ethical Review Committee of the University of Benin.

Response: The consent to participate was obtained from the interviewees at the time of recruitment.

The ethnical approval number has been included in the manuscript. Thank you.
